# A Simple, Rapid and Sensitive FRET Assay for Botulinum Neurotoxin Serotype B Detection

**DOI:** 10.1371/journal.pone.0114124

**Published:** 2014-12-01

**Authors:** Jiubiao Guo, Ci Xu, Xuechen Li, Sheng Chen

**Affiliations:** 1 Department of Applied Biology and Chemical Technology, The Hong Kong Polytechnic University, Hung Hom, Kowloon, Hong Kong SAR; 2 Department of Chemistry, University of Hong Kong, Pokfulam, Hong Kong; University of Wisconsin, Food Research Institute, United States of America

## Abstract

Botulinum neurotoxins (BoNTs), the most potent naturally-occurring neurotoxins known to humans, comprise seven distinct serotypes (BoNT/A-G), each of which exhibits unique substrate specificity. Many methods have been developed for BoNT detection, in particular for BoNT/A, with various complexity and sensitivity, while substrate based FRET assay is considered as the most widely used approach due to its simplicity and sensitivity. In this study, we designed a vesicle-associated membrane protein 2 (VAMP2) based FRET assay based on the understanding of the VAMP2 and light chain/B (LC/B) interactions in our previous studies. The current design constituted the shortest peptide, VAMP2 (63–85), with FRET dyes (EDAN and Dabcyl) labelled at position 76 and 85, respectively, which showed minimal effect on VAMP2 substrate catalysis by LC/B and therefore enhanced the sensitivity of the assay. The FRET peptide, designated as FVP-B, was specific to LC/B, with a detection sensitivity as low as ∼20 pM in 2 h. Importantly, FVP-B showed the potential to be scaled up and used in high throughput screening of LC/B inhibitor. The currently developed FRET assay is one of the most economic and rapid FRET assays for LC/B detection.

## Introduction

Botulinum neurotoxins (BoNTs), the most potent protein toxins identified to date, cause food-borne, wound and infant botulisms [1]. There are seven different BoNT serotypes, designated as A to G, and more than 30 different subtypes being identified so far [2], [3]. Although large-scale outbreak of botulism rarely occurs nowadays, sporadic cases of natural botulisms and medical emergencies due to clinical uses of BoNTs are still posing a threat to human health [4], [5], [6]. Most importantly, due to its potency and ease of distribution, BoNT is listed as one of the six most dangerous bioterrorist threats by the US Centres for Disease Control and Prevention (CDC) (www.bt.cdc.gov/agent/agentlist-category.asp).

BoNTs are 150-kDa single chain proteins that are activated by proteolysis to generate disulfide-linked di-chain proteins. BoNTs are typical A-B toxins that comprise three independent domains: a 50 kDa N-terminal light chain that is responsible for its enzymatic activity and zinc-dependent proteolysis; a 100 kDa C-terminal heavy chain that is involved in receptor binding and cellular uptake and composed of a translocation domain and a receptor binding domain [7], [8], [9]. BoNTs undergo a four-stage intoxication process when intoxicating cells: receptor binding, internalization, membrane translocation and cleavage of substrates [10], [11], [12]. The driving force in mammalian neuronal exocytosis process is the formation of complexes between the family of soluble N-ethylmaleimide-sensitive factor attachment protein receptors (SNAREs): the vesicle SNARE VAMP-2, the plasma membrane SNARs, SNAP25 and syntaxin 1 [13], which are the targets of BoNTs. Serotypes B, D, F and G cleave VAMP-2, serotypes A and E cleave SNAP25, and serotype C cleaves both SNAP25 and syntaxin 1 [7]. Release of neurotransmitter will be blocked upon the cleavage of any of the aforementioned SNARE proteins, leading to the classical paralytic symptoms of botulism.

For BoNT/A, the estimated lethal dosage for humans is 1µg/kg in the case of oral administration [1]. If diagnosed before the onset of symptoms, botulism can be effectively treated immunologically by using an equine trivalent antitoxin (www.bt.cdc.gov/agent/agentlist-category.asp). Early BoNT detection is critical to timely treatment of botulism. Currently, the “golden standard” for BoNT detection in culture, serum and food samples is mouse bioassay. It has a serotype and subtype dependent sensitivity of between 10-100 pg/ml [14], [15], and can detect all serotypes and subtypes both in their free and complex forms. However, it is time-consuming [16], unable to be scaled up and often arouses serious ethical concern, prompting a need to develop alternative assays to replace the mouse bioassay. PCR-based techniques that aim at detecting *bont* genes by conventional or quantitative amplification reactions, with detection limit of 10^3^–10^5^ genome equivalents (GE) per ml, have been developed [17], [18], [19], [20]. Mass spectrometry is a powerful tool in detecting different BoNT serotypes unambiguously [21], [22], [23], [24], [25], an amino acid substitution database has been established by Barr and co-workers, allowing for identification of multiple BoNT/B subtypes [3]. By far, the most commonly employed methods for BoNT detection in vitro is ELISA (enzyme-linked immunosorbent assay)-based technologies, which exhibit high sensitivity, simplicity, and robust performance [15], [26], [27], [28], [29]. Since the identification of the substrates of BoNTs, substrate based activity assays of BoNTs have been developed and improved, displaying the serotype-specific proteolytic cleavage of SNAREs [30]. The combination of the endopeptidase assay with FRET (Förster resonance energy transfer) that utilizes fluorescence donor and fluorescence acceptor (or quencher) makes it very a powerful and sensitive BoNT detection method [31], [32]. Substrate based FRET assay has the advantages of simplicity, rapidity, cost effectiveness and readiness for scale up, yet the detection sensitivity was shown to be very low due to the reduced activity of FRET peptide.

In the current work, we developed a VAMP2 based FRET assay for BoNT/B detection based on the understanding of the interactions between VAMP2 and LC/B. Previous works have identified the minimal substrate, VAMP2(63∼85), for LC/B optimal substrate cleavage [33] and the contribution of each residue in this fragment of VAMP2 to LC/B substrate hydrolysis. We then designed the minimal FRET peptide with the modification resulting in minimal effect on LC/B cleavage. This assay is one of the most economic and rapid FRET assays for LC/B detection.

## Experimental Section

### Recombinant BoNT LC purification

Recombinant LC/B protein was purified as previously described [33]. Other BoNT LCs including LC/A (1–425), LC/E (1–408), LC/F (1–446), LC/D (1–442) and LC/TeNT-(1–436) were expressed and purified as described previously [33], [34], [35], [36].

### Fluorogenic peptide design

**Figure 1:**
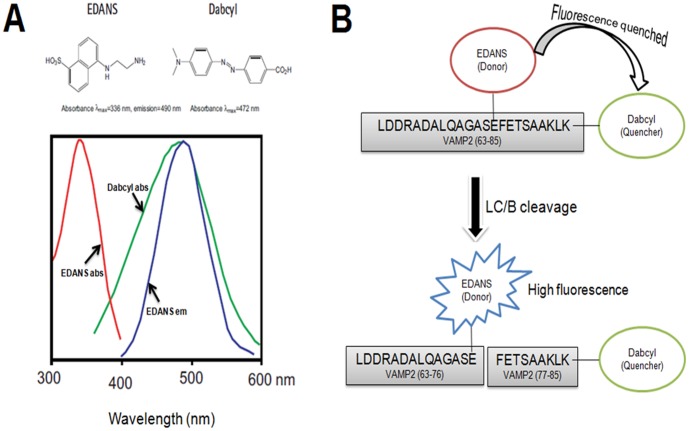
Spectral properties of EDANS-Dabcyl pair [45] and the flowchart of the experimental design. (A) EDANS-Dabcyl is a widely used donor-quencher pair. The optimal absorbance and emission wavelengths of EDANS are λ_abs_ = 336 nm and λ_em_ = 490 nm respectively, and for Dabcyl, the maximum absorbance wavelength is λ_abs_ = 472 nm, which, to a large extent, overlap with the emission spectra of EDANS. When they are in a close proximity (10–100 Å), the energy emitted from EDANS will be quenched by Dabcyl, resulting in low or no fluorescence; when they are separated upon substrate cleavage, for example in this design, the fluorescence will increase. Hence from the fluorescence intensity change, the enzyme could be detected continuously and directly. (B) Based on the principle of FRET and our previous study, we chose the optical LC/B cleavage length of VAMP2 (63–85) as the linker between EDANS-Dabcyl.

Previous studies showed that the optimal substrate for LC/B was VAMP2 (63∼85). Mutational analysis data also showed that Q76 of VAMP2 contributed limited effect on the activity of LC/B [33]. Therefore, the FRET peptide was designed to include residues 63∼85 of VAMP2 with EDANS labelled L-Glu replaced Q76 and Dabcyl labelled Lys located at the C-terminus. The peptide sequence was as follow, Leu-Asp-Asp-Arg-Ala-Asp-Ala-Leu-Gln-Ala-Gly-Ala-Ser-L-**Glu(EDANS)-Phe**-Glu-Thr-Ser-Ala-Ala-Lys-Leu-L-Lys (Dabcyl) (**Figure 1B**). EDANS (L-glutamic acid g-[b-(5-naphthyl sulfonic acid)-ethylenediamine] ester) and Dabcyl (*N* ^ε^-dimethylaminophenyldiazobenzoyl) is a FRET pair (**Figure 1A**). EDANS served as fluorescent donor and Dabcyl acted as fluorescent dye. In this design, the FRET peptide showed limited fluorescent signal. Upon cleavage by LC/B, EDANS was separated from Dabcyl and fluorescent signal increased. The FRET peptide was named as FVP-B.

### Fluorogenic peptide synthesis

All commercial materials (Sigma-Aldrich, Fluka of USA and GL Biochem of China) were used without further purification. All solvents were reagent grade or HPLC grade (DUKSAN). Dry dichloromethane was distilled from calcium hydride (CaH_2_). All separations involved a mobile phase of 0.05% TFA (*v/v*) in acetonitrile (solvent A)/0.05% TFA (*v/v*) in water (Solvent B). HPLC separations were performed with a Waters HPLC system equipped with a photodiode array detector (Waters 2996), using a Vydac C18 column (5 µm, 300 Å, 4.6×150 mm) at a flow rate of 0.6 mL/min for analytical HPLC, and Vydac Prep C18 column (10 µm, 300 Å, 22×250 mm) at a flow rate of 10 mL/min for preparative HPLC. Low-resolution mass spectral analyses were performed with a Waters 3100 mass spectrometer.

The solid phase peptide synthesis was carried out manually using 2-Chlorotrityl chloride resin (loading 0.5 mmol/g). Peptides were synthesized under standard Fmoc/*t*-Bu protocols. The following Fmoc amino acids from GL Biochem were employed: Fmoc-Ala-OH, Fmoc-Arg(Pbf)-OH, Fmoc-Asn(Trt)-OH, Fmoc-Asp(O*t*Bu)-OH, Fmoc-Gln(Trt)-OH, Fmoc-Glu(EDANS)-OH, Fmoc-Glu(O*t*Bu)-OH, Fmoc-Gly-OH, Fmoc-Ile-OH, Fmoc-Leu-OH, Fmoc-Lys(Boc)-OH, Fmoc-Lys(DABCYL)-OH, Fmoc-Met-OH, Fmoc-Phe-OH, Fmoc-Ser(*t*Bu)-OH, Fmoc-Thr(*t*Bu)-OH. Fmoc removal was executed using a solution of 20% piperidine in dimethylformamide (DMF) at room temperature for 30 min. Coupling of Fmoc protected amino acid units was carried out by activation with (*O*-(7-azabenzotriazol-1-yl)-*N*,*N*,*N*′,*N*′-tetramethyluroniumhexafluorophosphate (HATU) using N,N-diisopropylethylamine (DIPEA) in DMF at room temperature for 40 min. The Fmoc amino acids (2.0 equiv), HATU (2.0 equiv) and DIPEA (5.0 equiv) were dissolved in DMF and subsequently mixed with the resin manually. This procedure was repeated twice for each coupling. Upon completion of synthesis, the peptide resin was subjected to a cleavage of cocktail (TFA/*i*Pr_3_SiH/H_2_O, 95/2.5/2.5, *v/v/v*) for 2 h. The resin was filtered and the combined filtrates were blown off under a stream of condensed air. The crude product was triturated with cold diethyl ether to give a white suspension, which was centrifuged and the ether subsequently decanted. The remaining solid was purified by HPLC.

### Proteolytic activities of LC/B and other LCs on FRET peptide

All LCs used in this study were quantified by SDS-PAGE using BSA standards. The FVP-B was prepared in 21 mM stock in DMSO, aliquoted and stored at −20°C. It was diluted in reaction buffer (10 mM Tris-HCl, 20 mM NaCl, pH 7.9) prior to the assay. For LC/B activity assay, certain amounts of FVP-B were mixed with different concentrations of LC/B and incubated in 500 µl of reaction buffer in eppendorf tubes at 37°C for different time courses. The reaction mixture was transferred to a quartz fluorescence cuvette (Sigma-Aldrich Co. LLC. USA). Fluorescent intensity was scanned using LS-55 Fluorescence Spectrometer (PerkinElmer Inc. Massachusetts, USA), with the following parameters set: excitation: 336 nm (slit: 10 nm), emission: 380-650 nm (slit: 10 nm), speed: 100 nm/min. For EDTA inhibition assays, different concentrations of EDTA were incubated with LC/B and FVP-B in the similar assay as described above, but detected both in fluorescence cuvette and black 96 well plate (PerkElmer, USA). All the data were repeated at least three times.

### LC/MS analysis of FVP-B cleavage by LC/B

200 nM LC/B and 8.4 µM FVP-B were mixed in reaction buffer to a final volume of 500 µl and incubated at 37°C for 1 h. LC/MS analysis was then carried out on an Agilent 6540 Ultra High Definition Accurate-Mass Q-TOF (Agilent-Technologies Inc., Wilmington, United States of America) equipped with an Agilent 1290 Infinity binary LC system. A 2 µl portion of sample was injected into a C18 reverse column (Agilent Zorbax SB-C18, 2.1×100 mm, 1.8 µm). The mobile phase solvent system included solvent A, Milli-Q water with 0.1% formic acid, and solvent B, acetonitrile with 0.1% formic acid. The sample was firstly desalted with 5% solvent B at a flow rate of 0.3 ml/min for 3 min, eluted with a 17 min linear gradient of 5% to 90% solvent B at a flow rate of 0.3 ml/min. The mass spectrometer was operated in positive electrospray ionization mode. Other instrumental parameters were set as follow: capillary voltage: 3500 V, nozzle voltage: 1000 V, fragmentor voltage: 175 V, skimmer: 65 V, octopole RF: 750 V.

## Result

### LC/B specificity to FVP-B

**Figure 2:**
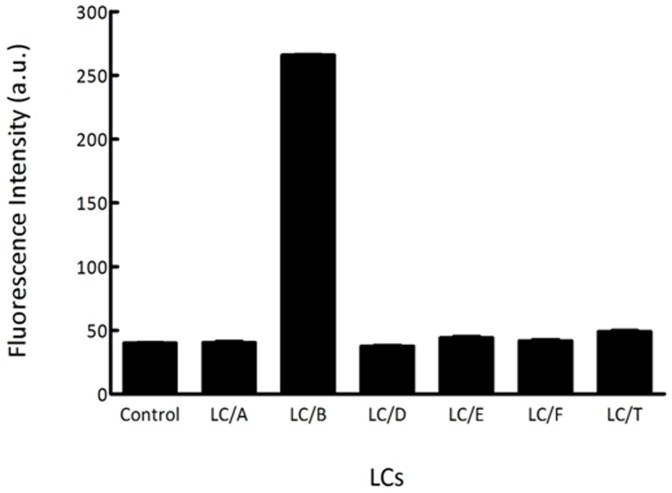
The specificity of the synthesized FVP-B. LCs (200 nM each) were mixed with 8.4 µM FVP-B in reaction buffer to a final volume of 500 µl, incubated at 37°C for 1 h, fluorescent intensity was measured by a Fluorescence Spectrometer in quartz cuvette. The data obtained were processed with GraphPad Prism.

The FRET assay is based on the detection of continuous signal increase resulting from the hydrolysis of FVP-B to separate quencher Dabcyl from the fluorescent donor EDANS. To verify the specificity of FVP-B to LC/B, FVP-B was used to test the cleavage by different LCs of BoNTs. In this assay condition where 200 nM each of the LCs were mixed with 8.4 µM FVP-B in reaction buffer in 500 µl reaction volume for 1 h incubation at 37°C, the fluorescence intensity of each reaction was quantified. The negative control was performed exactly the same as other reactions, but without adding the LCs in the reaction. **Figure 2** showed the fluorescent intensity of each reaction and the negative control. The result showed that dramatic increase of fluorescence intensity could be seen after incubating LC/B with FVP-B, whereas incubation of other LCs with FVP-B did not produce dramatic increase of fluorescence intensity, suggesting that FVP-B was specific to LC/B cleavage **(** **Figure 2** **)**.

**Figure 3:**
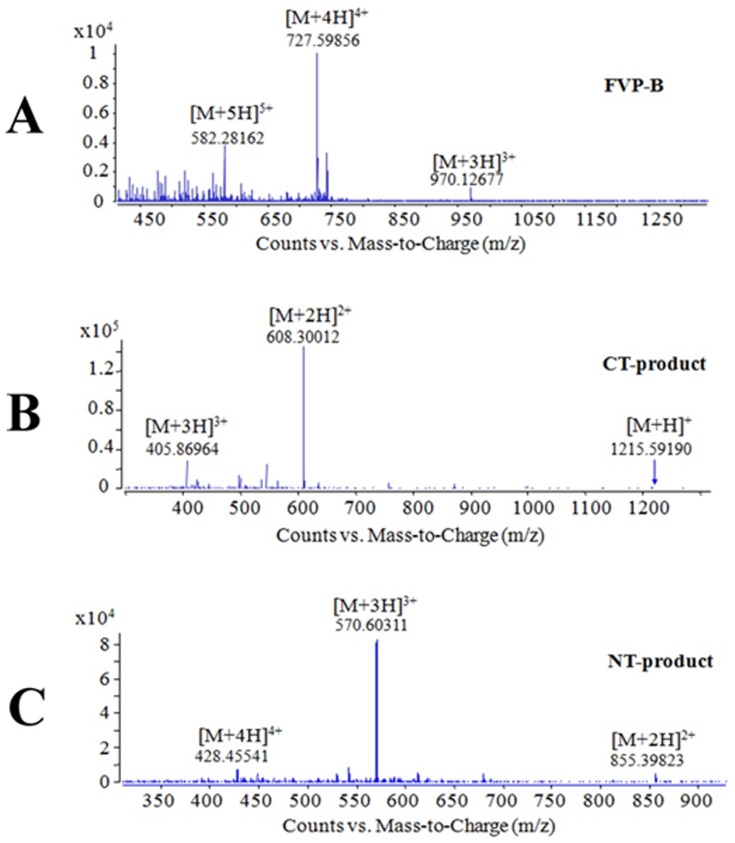
LC/MS analysis of specific cleavage of FVP-B by LC/B. Mixture sample of FVP-B (8.4 µM) and LC/B (200 nM) from Figure 2 was analysed by LC/MS to detect the full length FVP-B (A), CT-product (B) and NT-product (C).

The mixture sample of LC/B and FVP-B was then analysed by LC/MS to prove the specific cleavage of FVP-B by LC/B. As shown in **Figure 3**, the FVP-B (**Figure 3A**), C-terminal product of cleaved FVP-B (CT-product, **Figure 3B**) and N-terminal product of cleaved FVP-B (NT-product, **Figure 3C**) could be detected. The LC/MS results also proved that the sum of the molecular weights (M_r_) of CT-product (M_r_ = 1214.6) and NT-product (M_r_ = 1708.8) was about 2905.4 (with a H_2_O molecular deducted), which was the same as that of FVP-B (M_r_ = 2906.4), indicating that LC/B could specifically cleave FVP-B at the scissile bond.

### FVP-B assay for LC/B activity detection

**Figure 4:**
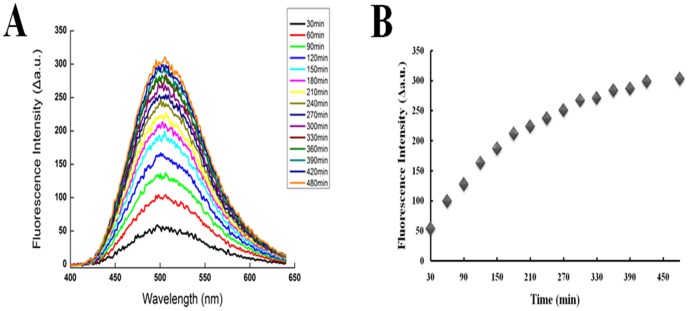
The feasibility of the synthesized FVP-B and our developed assay. (A) 228 nM purified LC/B was mixed with 8.4 µM FVP-B in reaction buffer to a final volume of 500 µl. Reaction was incubated at 37°C and the fluorescent intensity was measured in quartz cuvette every 30 min. The total duration of measurement was 8 h. The data were processed by Origin85. (B) The fluorescent intensity peak at 502 nm was selected to represent the trend of the whole data. The data were processed with Excel.

**Figure 5:**
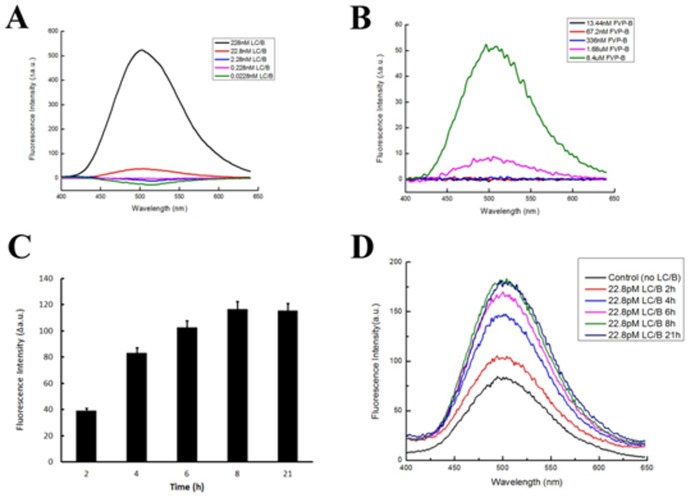
Optimization of the developed assay system. To optimize the assay and test the sensitivity of our detection assay, ten-fold dilution of LC/B was performed with the concentration of FVP-B fixed at 8.4 µM (A); five-fold dilution of FVP-B, with the LC/B concentration fixed at 22.8 nM (B); the fluorescent intensity change at 502 nm of further diluted LC/B to 22.8 pM with 1.68 µM FVP-B and extended incubation time (C); and a spectrum representative of 22.8 pM LC/B incubated with 1.68 µM FVP-B for extended time span (D). Viewing from the data, the detection sensitivity of the developed assay is about 22.8 nM of LC/B within 30 min, but the sensitivity can be improved to 22.8 pM of LC/B with 2 h incubation. The data were processed by Origin85 and Excel.

The effect of the cleavage of FVP-B by LC/B over a specific time period was determined. Firstly, 228 nM LC/B was mixed with 8.4 µM FVP-B in reaction buffer in 500 µl reaction system and incubated at 37°C. The fluorescent intensity between 400 and 650 nm was measured every 30 min (except the last 1 h) over 480 min assay time. After 30 min incubation, dramatic increase of fluorescence intensity could be observed. The signal increased steadily until reaching a steady stage at 480 min. Sharp increase of fluorescent intensity continued for at least 120 min and the rate of fluorescence increase slowed down after about 180 min and finally reached to a steady state at 480 min (**Figure 4A**). LC/B velocity curve was generated by recording the rate of increase in fluorescence intensity at 502 nm. The curve clearly indicated that fluorescence intensity increased over time (**Figure 4B**). To optimize the assay and test the sensitivity of FVP-B for LC/B detection, the LC/B concentration was titrated. First, FVP-B concentration was fixed at 8.4 µM and 10-fold dilution of LC/B concentration was performed. A dramatic fluorescent intensity increase was shown at LC/B concentration of 228 nM. Obvious fluorescent intensity increase can be seen at LC/B concentration of 22.8 nM, yet no fluorescent intensity change can be observed at lower concentration of LC/B (**Figure 5A**). Secondly, LC/B concentration was fixed at 22.8 nM and 5-fold FVP-B titration was performed. Significant fluorescent intensity increase can be seen when FVP-B concentration was at 8.4 µM, and relatively obvious fluorescent change can be seen at 1.68 µM of FVP-B, but not at lower concentration (**Figure 5B**). However, when the concentration of FVP-B was fixed at 1.68 µM, we further diluted the LC/B to 22.8 pM with extended incubation time. After 2 h incubation at 37°C, obvious fluorescent intensity change can be detected, and 4 h incubation showed even dramatic fluorescent intensity change, but longer incubation time up to 8 h cannot produce any further increase in fluorescent intensity. The fluorescent intensity was shown to be very stable (**Figure 5C and 5D**). Taken together, the assay optimization results showed that the detection sensitivity of the developed assay is about 20 nM of LC/B within 30 min, but the sensitivity can be improved to ∼20 pM of LC/B with 2 h incubation. Additionally, we have tested the effect of different concentrations of zinc on LC/B activity and did not observe any association between zinc ion and LC/B activity (data not shown).

**Figure 6:**
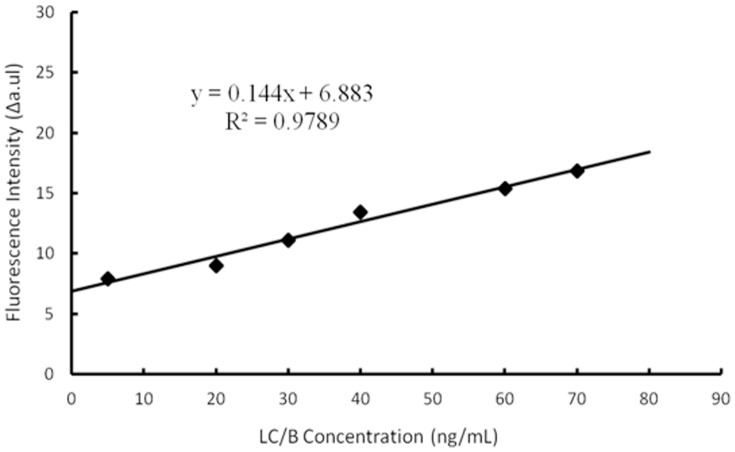
Limit of detection of the developed LC/B detection system. Serial LC/B concentrations were incubated with 8.4 µM FVP-B in 500 µl reaction volume for 1 h incubation at 37°C. The fluorescence intensities were potted versus the concentration of LC/B. For each concentration, at least five replicates were carried out. LOD  = 3*S/k (S means standard deviation of negative control, k means slope).

To further illustrate the sensitivity of the developed system, various amounts of LC/B were incubated with 8.4 µM FVP-B in 500 µl reaction volume for 1 h incubation at 37°C. The limit of detection (LOD) was determined at about 4.1 ng/mL (**Figure 6**).

### FVP-B as LC/B inhibitor screening assay

**Figure 7:**
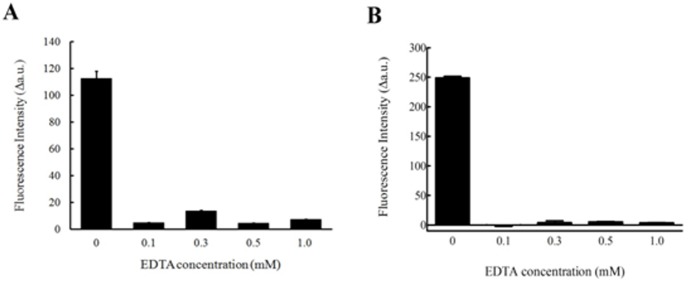
The inhibitory effect of EDTA on LC/B. 228 nM of LC/B was mixed with 8.4 µM FVP-B with reaction buffer to a final volume of 500 µl, incubated at 37°C for 1 h, and then the fluorescent intensity was measured by a Fluorescence Spectrometer in quartz cuvette (A); 100 µl reaction volume, which contained 228 nM LC/B with 8.4 µM FVP-B, were carried out in 96 well plate after 1 h incubation at 37°C (B). For simplicity, the fluorescent intensity peak at 502 nm was selected. The data obtained were generated from at least three times repeats, and then processed with Excel and GraphPad Prism, with the negative control data subtracted.

To prove that this assay can be scaled up for BoNT/B inhibitor screening, EDTA was used as an inhibitor of LC/B to test the validity of the assay. Different concentrations of EDTA were tested in the assay, where 228 nM of LC/B and 8.4 µM FVP-B were mixed and incubated for 1 h at 37°C. The fluorescent intensity was recorded at 502 nm, measured both in fluorescent cuvette (500 µl reaction volume, **Figure 7A**) and 96 wellplate (100 µl reaction volume, **Figure 7B**). At 0.1, 0.3, 0.5 and 1 mM EDTA, fluorescent intensity remained at the same level as control (no LC/B added), while at 0 mM of EDTA, the fluorescent intensity showed dramatic increase (**Figure 7A and 7B**). The data indicated that FVP-B based assay is a useful tool for high throughput LC/B inhibitor screening.

## Discussion

BoNTs are the most potent naturally-occurring toxins known. Although BoNT/B is not as clinically important as BoNT/A in term of its ability to cause human botulism and its usefulness in various therapeutic processes, BoNT/B is still frequently linked to human botulism, in particular infant botulism. Data from CDC showed that 58.4% cases of infant botulism (387 out of 663) were attributed to BoNT/B during a 2001 to 2007 surveillance [37]. In addition, BoNT/B is also used as human therapy to treat dystonia in addition to BoNT/A. Moreover, because of the many ethical and legal concerns over the standard mouse bioassay, an alternative, simple and sensitive detection method is urgently needed to either detect trace of BoNT/B in food or for designing new potent inhibitor to neutralize the toxin. Recently, several new methods have been reported to detect BoNT/B with higher sensitivity in the picomolar to femtomolar ranges [38]; these include common methods like ELISA with comparable sensitivity as mouse bioassay [37], [39], and fast detection methods such as micromachined BoNT/B detection sensor for BoNT/B detection within minutes, but with lower sensitivity [40]. However, all these methods need complicated procedures, expensive apparatus and are difficult to be scaled up. FRET coupled LC/B detection methods have been reported before [31], [41]. However, the synthesized peptides were much longer (either contained VAMP2 (residues 55–94) or VAMP2 (residues 60–94)) than the peptide reported here. Most importantly, some have mutated or replaced the residue F^77^ of VAMP2, which will dramatically reduce the efficiency of LC/B to cleave this substrate according to our previous data that the P1′ site played a very important role in enzyme catalysis: the mutant VAMP2 (F^77^A) reduced the LC/B cleavage efficiency to more than 320-fold [33]. In addition, there are several versions of FRET based peptides available in market (List Biological Laboratories, INC. USA), and one of them has been claimed to exhibit a LOD as low as 140 pg/mL after 5 h digest or 8 pg/mL after 24 h digest. Christine Anne *et al* reported a fluorogenic assay system for BoNT/B activity determination with a 3.5 pg/mL detection sensitivity after 4 h incubation time [31]. However, it is questionable that there is no effect of BoNT/B cleavage of peptide, as claimed by the authors, by replacing the native phenylalanine in position 77 by a *p*-nitrophenylalanine. Moreover, the cost of the claimed high sensitivity is the longer incubation time, 4 h or even longer, when compared to the present assay which needs as short as 1 h incubation to reach a 4.1 ng/mL LOD. In addition, the different reaction conditions, buffer system, sensitivity of spectrometer could also affect the LOD of known commercial systems. The current assay seems more stable, reproducible and more economic due to its shortest length compared to the commercial kits.

In this study, we developed a simple, fast and sensitive VAMP2 based FRET peptide for LC/B detection. The detection sensitivity is about 20 pM of BoNT/B with 2 h incubation. FRET based BoNTs detection method has been proven to be very useful in both clinical application and research. SNAPtide, a commercial 13mer FRET peptide developed for BoNT/A detected has been widely used in BoNT/detection and inhibitor screening in many researches [42], [43], [44]. However, due to the lower cleavage efficiency by BoNT/A, the role of SANPtide as high throughput assay was limited. Since FVP-B is designed based on the optimal substrate for BoNT/B, the FRET peptide we developed has very similar cleavage efficiency by BoNT/B as natural substrate VAMP2. We have designed another version of FRET based peptide, the positions modified were A74 and T79, the single-letter sequence is 63-L D D R A D A L Q A G E-(Edans) S Q F E K-(Dabcyl) S S A K L K-85. Based on the previous investigation, the mutation or modification on 74 or 79 has less effect on LC/B cleavage than that of 76 or 85 (the two sites modified in the present FVP-B peptide) [33], but it showed much lower sensitivity and lower LC/B cleavage efficiency than FVP-B (data not shown). Therefore, FVP-B based assay represents a good FRET assay for BoNT/B detection and also proved to be useful for high throughput BoNT/B inhibitor screening.
